# Genetic polymorphism and natural selection of circumsporozoite surface protein in *Plasmodium falciparum* field isolates from Myanmar

**DOI:** 10.1186/s12936-018-2513-0

**Published:** 2018-10-12

**Authors:** Hương Giang Lê, Jung-Mi Kang, Mya Moe, Hojong Jun, Thị Lam Thái, Jinyoung Lee, Moe Kyaw Myint, Khin Lin, Woon-Mok Sohn, Ho-Joon Shin, Tong-Soo Kim, Byoung-Kuk Na

**Affiliations:** 1 Department of Parasitology and Tropical Medicine and Institute of Health Sciences, Gyeongsang National University College of Medicine, Jinju, 52727 Republic of Korea; 2 BK21Plus Team for Anti-aging Biotechnology and Industry, Department of Convergence Medical Science, Gyeongsang National University, Jinju, 52727 Republic of Korea; 3 Department of Medical Research Pyin Oo Lwin Branch, Pyin Oo Lwin, Myanmar; 4 Department of Tropical Medicine and Inha Research Institute for Medical Sciences, Inha University College of Medicine, Incheon, 22212 Republic of Korea; 5 Department of Microbiology, Ajou University College of Medicine, Suwon, 16499 Republic of Korea

**Keywords:** *Plasmodium falciparum*, Circumsporozoite protein, Genetic polymorphism, Natural selection, Myanmar

## Abstract

**Background:**

*Plasmodium falciparum* circumsporozoite protein (PfCSP) is one of the most extensively studied malaria vaccine candidates, but the genetic polymorphism of PfCSP within and among the global *P. falciparum* population raises concerns regarding the efficacy of a PfCSP-based vaccine efficacy. In this study, genetic diversity and natural selection of PfCSP in Myanmar as well as global *P. falciparum* were comprehensively analysed.

**Methods:**

Blood samples were collected from 51 *P. falciparum* infected Myanmar patients. Fifty-one full-length PfCSP genes were amplified from the blood samples through a nested polymerase chain reaction, cloned into a TA cloning vector, and then sequenced. Polymorphic characteristics and natural selection of Myanmar PfCSP were analysed using the DNASTAR, MEGA6, and DnaSP programs. Polymorphic diversity and natural selection in publicly available global PfCSP were also analysed.

**Results:**

The N-terminal and C-terminal non-repeat regions of Myanmar PfCSP showed limited genetic variations. A comparative analysis of the two regions in global PfCSP displayed similar patterns of low genetic diversity in global population, but substantial geographic differentiation was also observed. The most notable polymorphisms identified in the N-terminal region of global PfCSP were A98G and 19-amino acid length insertion in global population with different frequencies. Major polymorphic characters in the C-terminal region of Myanmar and global PfCSP were found in the Th2R and Th3R regions, where natural selection and recombination occurred. The central repeat region of Myanmar PfCSP was highly polymorphic, with differing numbers of repetitive repeat sequences NANP and NVDP. The numbers of the NANP repeats varied among global PfCSP, with the highest number of repeats seen in Asian and Oceanian PfCSP. Haplotype network analysis of global PfCSP revealed that global PfCSP clustered into 103 different haplotypes with geographically-separated populations.

**Conclusion:**

Myanmar and global PfCSP displayed genetic diversity. N-terminal and C-terminal non-repeat regions were relatively conserved, but the central repeat region displayed high levels of genetic polymorphism in Myanmar and global PfCSP. The observed geographic pattern of genetic differentiation and the points of evidence for natural selection and recombination suggest that the functional consequences of the polymorphism should be considered for developing a vaccine based on PfCSP.

**Electronic supplementary material:**

The online version of this article (10.1186/s12936-018-2513-0) contains supplementary material, which is available to authorized users.

## Background

Malaria, caused by *Plasmodium* spp. infections, is one of the most significant life-threatening infectious diseases to humans worldwide. It accounted for more than 216 million cases and approximately 450,000 deaths across the globe in 2017 [1]. The Greater Mekong Subregion (GMS) including Myanmar has long been one of the most malarious regions in the world [2]. Among the countries in GMS, Myanmar has the highest malaria burden, accounting for an estimated 77% of malaria cases and approximately 79% of malaria deaths in the GMS [3]. In spite of recent decreases in malaria cases and deaths, malaria is still a major public health concern in Myanmar [4].

To date, there is no licensed vaccine against malaria, though many efforts and studies have been performed in order to develop effective vaccines. Various vaccine constructs based on diverse antigens from sexual and asexual stages of *Plasmodium falciparum* have been investigated. Among these, RTS,S, currently the most advanced malaria vaccine candidate [5, 6], is based on circumsporozoite protein of *P. falciparum* (PfCSP). RTS,S is comprised of a liposome-based adjuvant (AS01) and hepatitis B virus surface antigen (HBsAg) virus-like particles incorporating a portion of the PfCSP genetically fused to HBsAg. PfCSP is a dominant surface protein of sporozoites, and it plays a critical role in the invasion of hepatocytes by sporozoites [7–9]. PfCSP is divided into three distinct regions: a highly variable central repeat region flanked by a conserved N-terminal region and a C-terminal non-repeat region. The central repeat region, which has been recognized as a major target for antibody-mediated neutralization, is rich in Asn-Ala-Asn-Pro (NANP) tandem repeats and also contains a small number of Asn-Val-Asp-Pro (NVDP) motifs [10–12]. The C-terminal non-repeat region includes two polymorphic sub-regions, Th2R and Th3R, where T cell epitopes were identified. These regions show moderate polymorphisms which might have resulted from natural selection by the host immune system [13–15].

Recent genome sequencing studies have demonstrated that *P. falciparum* from different geographic regions have diverse genetic makeup [16, 17], which emphasizes the importance of comprehensive analysis of parasite genetic diversity and population structure in the global *P. falciparum* population. Indeed, most *P. falciparum* vaccine candidate antigens including PfCSP have been found to show various genetic and antigenic polymorphisms in global isolates [18], which can obstruct or reduce the efficacy of vaccines based on PfCSP. Therefore, understanding the genetic nature of vaccine candidate antigens in global *P. falciparum* isolates is critical for designing an effective vaccine. In this study, genetic polymorphism and natural selection of PfCSP in *P. falciparum* Myanmar isolates were analysed. A comparative analysis of global PfCSP was also performed in order to gain an in-depth understanding of the genetic makeup of PfCSP in the global *P. falciparum* population.

## Methods

### Blood samples

A total of 51 blood samples used in this study were collected from malaria patients infected with *P. falciparum* in Myanmar in 2015. The patients were selected in field surveys for malaria, which were conducted in towns and villages in Naung Cho, Pyin Oo Lwin, Tha Beik Kyin townships, and Mandalay in Upper Myanmar. Infections were diagnosed through microscopic examination of thin and thick blood smears. Finger-prick blood samples were taken from *P. falciparum* infected symptomatic patients prior to drug treatment and spotted on Whatman 3 MM filter paper (GE Healthcare, Maidstone, UK) for confirmation by polymerase chain reaction (PCR) targeting the 18S ribosomal RNA (rRNA) gene [4, 19], as well as for subsequent molecular analysis. The mean age of patients who donated the blood samples was 32.7 years-old and ranged between 13 and 57 years. Informed consent was obtained from all patients before blood collection. The study protocol was reviewed and approved by either the Ethics committee of the Ministry of Health, Myanmar (97/Ethics 2015) or the Biomedical Research Ethics Review Board of Inha University School of Medicine, Republic of Korea (INHA 15-013).

### Genomic DNA extraction and amplification of PfCSP

Genomic DNA was extracted from dried blood spots using the QIAamp DNA Blood Kit (Quiagen, Hilden, Germany) following the manufacturer’s protocol. The full-length region encoding PfCSP was amplified through a nested PCR method. The primers for the first round PCR were 5′-ATG ATG AGA AAA TTA GCT ATT TTA TCT GTT-3′ and 5′-CTA ATT AAG GAA CAA GAA GGA TAA TAC CAT-3′. The primers used for nested PCR were 5′-AGA AAA TTA GCT ATT TTA TCT GTT TCT-3′ and 5′-ACA AGA AGG ATA ATA CCA TTA TTA ATC-3′. Ex *Taq* DNA polymerase (Takara, Otsu, Japan) with proof-reading activity was used in all PCR amplification steps to minimize the nucleotide mis-incorporation. The following thermal cycling conditions were used for both amplifications: 94 °C for 5 min; and 30 cycles of 94 °C for 1 min, 52 °C for 1 min, and 72 °C for 1 min 30 s, followed by a final extension at 72 °C for 10 min. The PCR product was resolved on a 1.2% agarose gel, purified from gel, then ligated into the T&A cloning vector (Real Biotech Corporation, Banqiao City, Taiwan). Each ligation mixture was transformed into *Escherichia coli* DH5α competent cells and positive clones, with appropriate inserts screened by colony PCR. The nucleotide sequences of the cloned PfCSP were analysed through automatic DNA sequencing with M13 forward and M13 reverse primers by the Sanger method. Plasmids from at least two independent clones from each transformation mixture were sequenced in both directions so as to verify the sequence accuracy. The nucleotide sequences reported in this study have been deposited in the GenBank database under the accession numbers MF350670–MF350720.

### Sequence polymorphism analysis

The nucleotide and deduced amino acid sequences of PfCSP were analysed using EditSeq and SeqMan in the DNASTAR package (DNASTAR, Madison, WI, USA). The PfCSP sequence of the laboratory-adapted *P. falciparum* strain 3D7 (XM_001351086) was included in the alignment for comparison as a reference sequence. The values of segregating sites (S), the average number of pair-wise nucleotide differences (*K*), haplotype diversity (Hd), and nucleotide diversity (π) were calculated using DnaSP version 5.10.00 [20]. The π was also calculated on a sliding window plot of 10 bases with a step size of 5 bp in order to estimate the stepwise diversity across the sequences. In order to test the null hypothesis of neutrality of PfCSP, the rates of synonymous (dS) and non-synonymous (dN) substitutions were estimated and were compared using the Z-test (*P* < 0.05) in MEGA6 program [21] using Nei and Gojobori’s method [22] with the Jukes and Cantor (JC) correction of 1000 bootstrap replications. Tajima’s D test [23], Fu and Li’s D and F statistics analysis [24] were performed using DnaSP ver. 5.10.00 [20] in order to evaluate the neutral theory of natural selection. The recombination parameter (R), which included the effective population size and probability of recombination between adjacent nucleotides per generation, and the minimum number of recombination events (Rm) were analysed using DnaSP ver. 5.10.00 [20].

### Genetic diversities of PfCSP among global *Plasmodium falciparum* isolates

The genetic diversities of PfCSP among global *P. falciparum* isolates were analysed. The PfCSP sequences used in this study were from Thailand, Philippines, Vietnam, India, Iran, Papua New Guinea, Vanuatu, Solomon Islands, Kenya, Cameroon, Ghana, Tanzania, Senegal, Gambia, Brazil, and Venezuela (Additional file 1: Table S1). These sequences cover full-length or partial portions of PfCSP. Genetic polymorphism and tests of neutrality were calculated for each population using DnaSP ver. 5.10.00 [20] and MEGA6 [21] as described above. A logo plot was constructed for each PfCSP population in order to analyse polymorphic patterns of the C-terminal non-repeat region in global PfCSP using the WebLogo program (https://weblogo.berkeley.edu/logo.cgi). In order to investigate the genetic relationships among global PfCSP haplotypes, the haplotype network for 817 full-length sequences of PfCSP from Myanmar and other countries listed above was analysed using NETWORK version 5.0.0.3 with the Median joining algorithm [25].

## Results

### Amplification of Myanmar PfCSP

Fifty-one full-length PfCSP were successfully amplified from the 51 blood samples analysed in this study. As expected, size variations were observed in the amplified PfCSP. The approximate sizes of amplified products varied from 0.9 to 1.3 kb, which was mainly caused by differences in the number of tandem repeats in the central repeat region.

### Genetic polymorphisms in the N-terminal region of Myanmar and global PfCSP

**Fig. 1:**
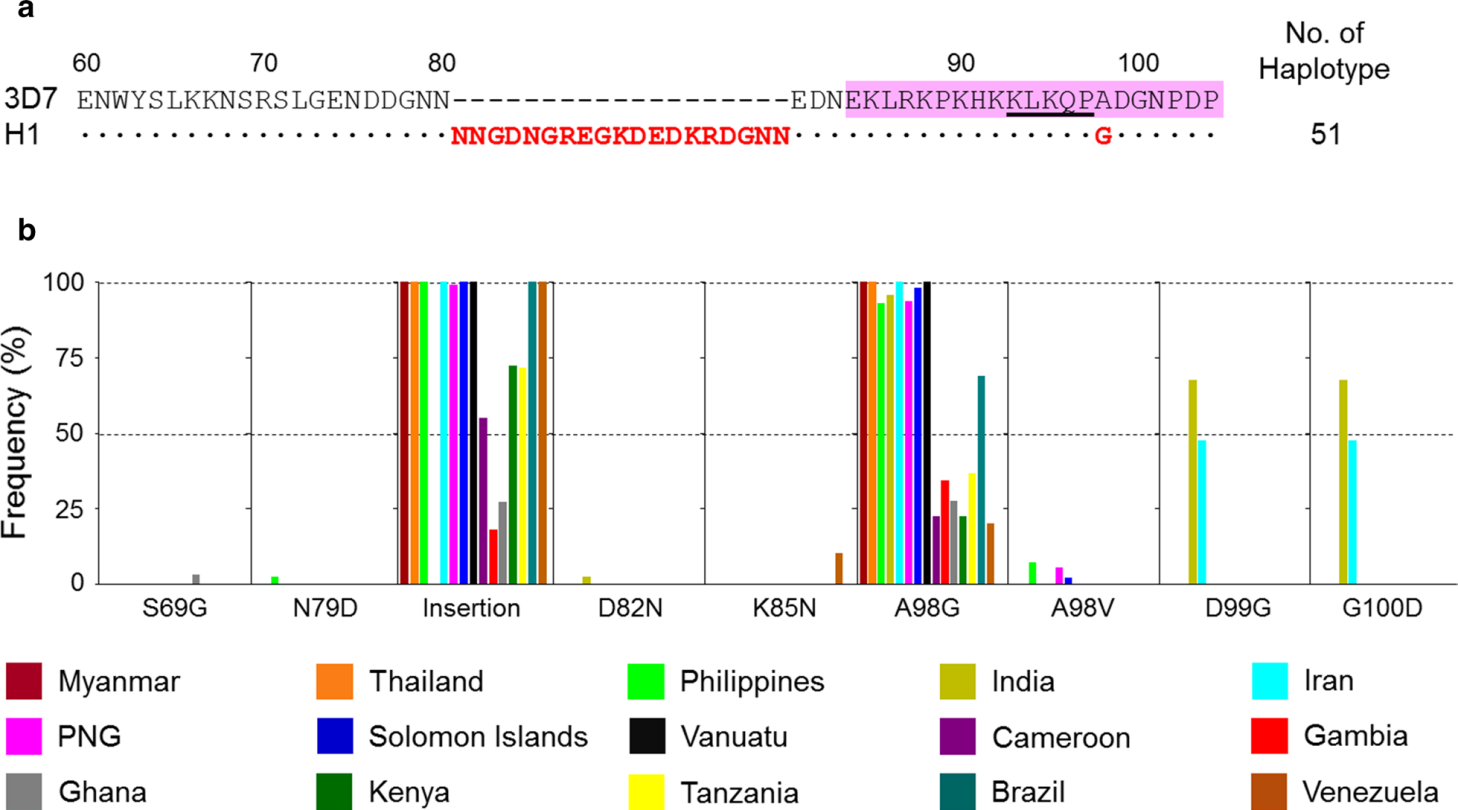
Genetic polymorphisms of the N-terminal non-repeat region in Myanmar and global PfCSP. **a** Polymorphic pattern of the N-terminal non-repeat region in Myanmar PfCSP. Only a haplotype of the N-terminal non-repeat region was identified in 51 Myanmar PfCSP sequences. All Myanmar PfCSP had 19 amino acids length insertion (NNGDNGREGKDEDKRDGNN) in the middle of the region. The dots represent residues identical to the reference sequence of 3D7 (XM_001351086). The dashes represent gaps to maximize the alignment. The predicted T-cell epitope region was shaded with pink. The conserved motif (KLKQP) involved in the sporozoite invasion of mosquito salivary gland and in binding to hepatocytes prior to invasion was underlined. **b** Polymorphic patterns of the N-terminal non-repeat region in global PfCSP. The N-terminal non-repeat region of PfCSP was largely conserved in global PfCSP. Amino acid changes at seven positions and insertion of 19 amino acids length (NNGDNGREGKDEDKRDGNN) were the major variations observed in global PfCSP. The frequencies of the amino acid polymorphisms and insertion observed in global PfCSP differed by geographical origins. *PNG* Papua New Guinea

The N-terminal non-repeat region was highly conserved in Myanmar PfCSP. Only a haplotype with a 57 bp (encoding 19 amino acids of NNGDNGREGKDEDKRDGNN) insertion in the middle portion of the region compared to the 3D7 reference sequence (XM_001351086) was identified (Fig. 1a). A comparative analysis of the N-terminal non-repeat region in global PfCSP also showed that the region is relatively well-conserved in global PfCSP. The 19 amino acids length insertion was the major variation observed in global PfCSP, but the frequency of this insertion in global PfCSP differed according to geographical origin. Asian PfCSP, that is, from Myanmar, Thailand, Philippines, and Iran with the exception of India, showed the 19 amino acids insertion in the region. Oceanian and South American PfCSP, that is, from PNG, Solomon Islands, Vanuatu, Brazil, and Venezuela, also showed a high frequency of the insertion, ranging from 98.9 to 100%. Meanwhile, the frequency was lower in African PfCSP, specifically in Cameroon (55.0%), Gambia (18.0%), Ghana (27.0%), Kenya (72.2%), and Tanzania (71.6%) (Fig. 1b). Amino acid polymorphisms were also found at seven positions (S69G, N79D, D82N, K85N, A98G/V, D99G and G100D) in global PfCSP. A98G was commonly identified in global PfCSP, but its frequency varied among global PfCSP. The overall frequency was high (up to 92%) in Asian and Oceanian PfCSP, but low (less than 37%) in African and South American PfCSP, with the exception of Brazil (69.0%). Meanwhile, S69G, N79D, D82N, K85N, and A98V showed uneven geographic distributions and very low frequencies. D99G and G100D were identified only in Indian and Iranian PfCSP. Most amino acid polymorphisms identified in the N-terminal region of PfCSP were located in the predicted T cell epitope region, ^84^EKLRKPKHKKLKQPADGNPDP^104^. However, the conserved motif (KLKQP) that was involved in sporozoite invasion of mosquito salivary gland and in binding to hepatocytes prior to invasion [26] was well-conserved in all global PfCSP.

### Genetic polymorphisms in the central repeat region of Myanmar PfCSP

**Fig. 2:**
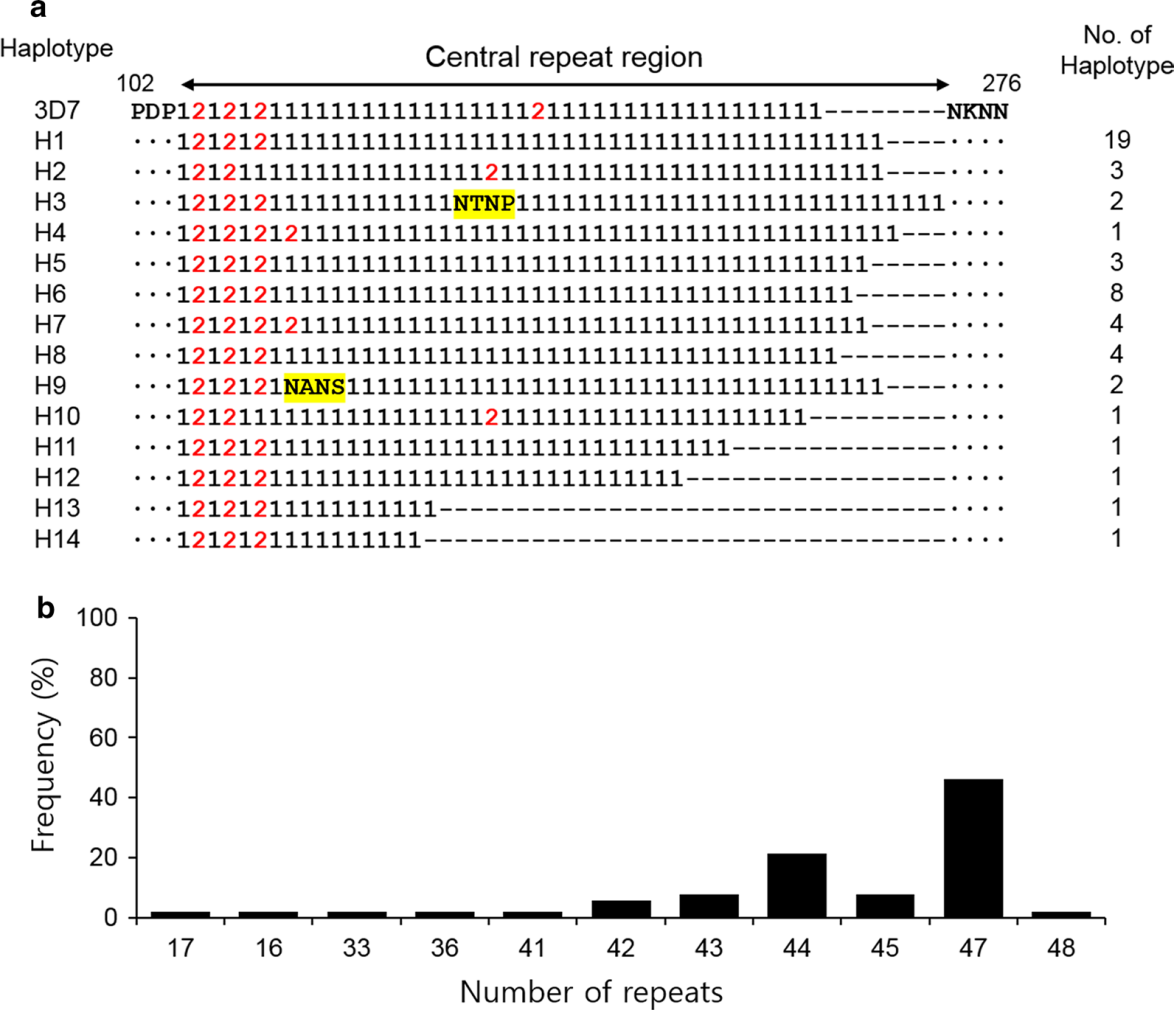
Genetic polymorphisms in the central repeat region of Myanmar PfCSP. **a** Polymorphic characters in the central repeat region of Myanmar PfCSP. Sequence alignment revealed that the central repeat region of Myanmar PfCSP showed polymorphic characters with 14 distinct haplotypes. Residues identical to those of the 3D7 PfCSP reference sequence (XM_001351086) are shown by dots. The dashes represent gaps introduced to maximize the alignment. The NANP repeat and NVDP repeat are indicated as one and two, respectively. The two new tetrapeptides, NTNP and NANS, are shaded with yellow. The number of haplotypes indicated the number of Myanmar PfCSP belonging to each haplotype. **b** Frequency of number of repeats in the central region among Myanmar PfCSP

A total of 14 unique haplotypes of Myanmar PfCSP were identified in amino acid levels (Fig. 2a). Two novel repeat allotypes, which encode NTNP and NANS motifs, were identified in two haplotypes (H3 and H9) of Myanmar PfCSP. Each haplotype of Myanmar PfCSP had different numbers of previously-known tetrapeptide repeats, NANP and NVDP motifs, ranging from 17 to 48. These different numbers of repeats resulted in size polymorphisms in the central repeat region among Myanmar PfCSP. Most Myanmar PfCSP had numbers of tetrapeptide repeats between 44 and 47 with a frequency of 76.4% (Fig. 2b).

### Polymorphic patterns of NANP repeats in global PfCSP

**Fig. 3:**
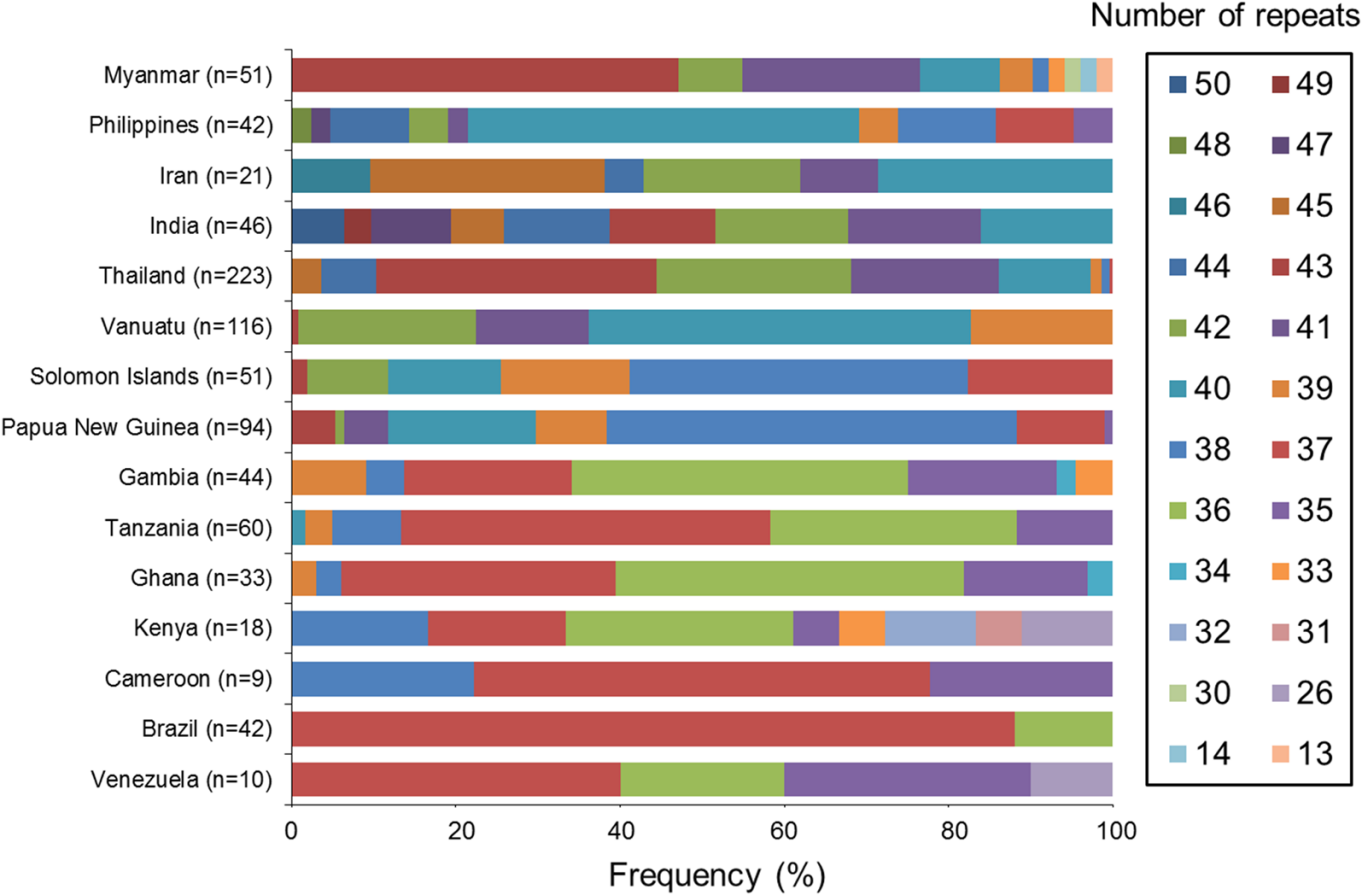
Frequency comparison of NANP repeats in the central repeat region of global PfCSP from different geographic regions. The numbers of NANP repeats differed in the global PfCSP population. In general, Asian PfCSP had a higher number of NANP repeats than African, Oceanian, and South American PfCSP

The numbers of NANP repeats in PfCSP populations from different geographical regions including Philippines, Iran, India, Thailand, Vanuatu, Papua New Guinea, Solomon Islands, Gambia, Tanzania, Ghana, Kenya, Cameroon, Brazil, and Venezuela (Additional file 1: Table S1) were analysed and compared with those of Myanmar PfCSP. Comparative analysis of global PfCSP revealed that the numbers of NANP repeats in global PfCSP differed by geographical origins (Fig. 3). Asian PfCSP had a high number of NANP repeats ranging from 40 to 43. Meanwhile, 36 and 37 NANP repeats were mainly observed in African and South American PfCSP. PfCSP from two Oceanian countries, Papua New Guinea and Solomon Islands, had 38 NANP repeats with a high frequency, whereas a higher number of repeats (40 repeats) was predominant in Vanuatu PfCSP.

### Polymorphic patterns in the C-terminal region of Myanmar and global PfCSP

**Fig. 4:**
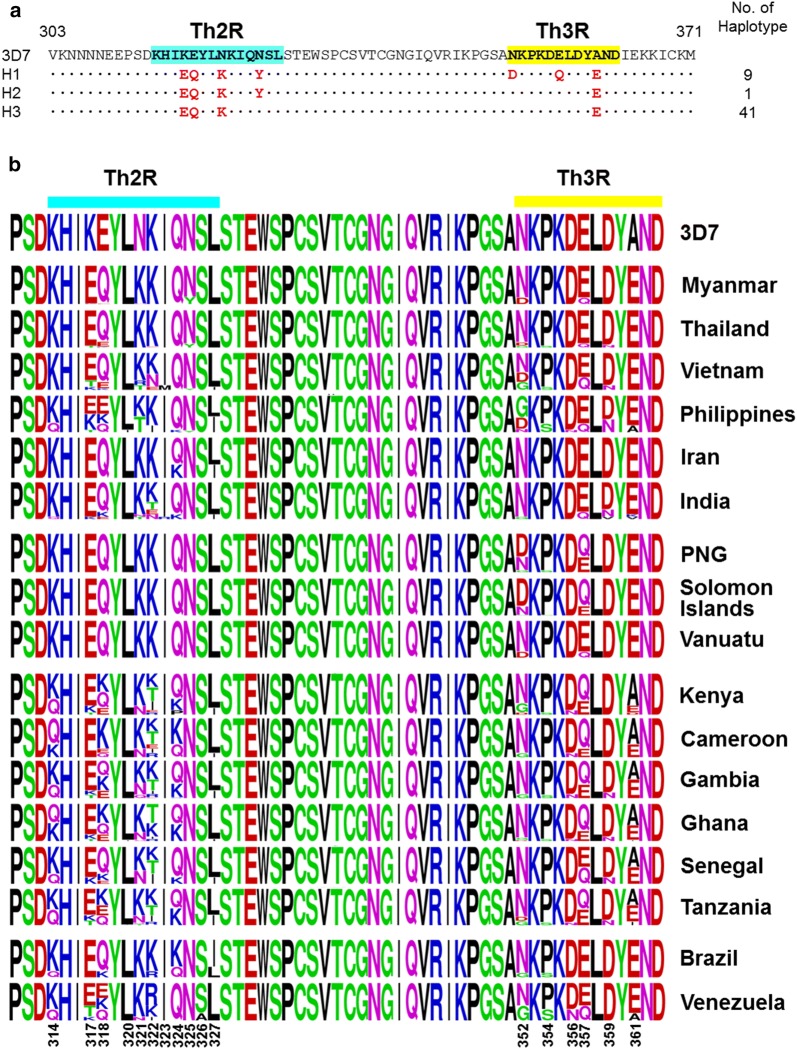
Polymorphic patterns of the C-terminal non-repeat region in Myanmar and global PfCSP. **a** Amino acid polymorphisms in the C-terminal non-repeat region of Myanmar PfCSP. The three different haplotypes (H1–H3) of the C-terminal non-repeat region were identified in Myanmar PfCSP. Th2R and Th3R regions are shaded with sky blue and yellow, respectively. Amino acid changes at particular amino acid positions are indicated as red. The number of haplotypes is the number of PfCSP sequences belonging to each haplotype. The dots represent residues identical to the reference sequence of 3D7 (XM_001351086). **b** Comparative analysis of polymorphic patterns of the C-terminal non-repeat region in global PfCSP. The pattern of amino acid changes differed by country or geographic continent, but most amino acid changes were concentrated in the Th2R and Th3R regions in global PfCSP. A logo plot was constructed for each PfCSP population using the WebLogo program. *PNG* Papua New Guinea

Three different haplotypes (H1–H3) were identified in the C-terminal non-repeat region of Myanmar PfCSP (Fig. 4a). The seven non-synonymous amino acid changes (K317E, E318Q, N321K, N325Y, N352D, E357Q and A361E) found in Myanmar PfCSP were located at the Th2R (^314^KHIKEYLNKIQNSL^327^) and Th3R (^352^NKPKDELDYAND^363^) T-cell epitope regions. Haplotype 3 was the most prevalent haplotype, accounting for 80.4% of the 51 Myanmar PfCSP sequences. The patterns of amino acid polymorphisms in global PfCSP were also assessed. Comparative analysis of polymorphic patterns of the C-terminal region in global PfCSP revealed that the region was relatively conserved in global PfCSP (Fig. 4b). However, complicated patterns of amino acid polymorphisms were also observed in the Th2R and Th3R regions. Compared to the 3D7 (XM_001351086) reference sequence, di-morphic or poly-morphic amino acid changes were identified at 17 positions in global PfCSP, all of which were observed in the Th2R (K314Q, K317E/T, E318Q/K, L320I, N321K/R/T/Q, K322N/E/T/I/R, I323M/R, Q324K/P, N325Y, S326A, and L327I) and Th3R (N352D/G, P354S, D356N, E357Q, D359N/V, and A361E/I/K/V/D) regions. The overall amino acid polymorphic patterns of these amino acids were more complex in African PfCSP than in PfCSP from other continents. The changes in K314Q, K322E/T/I/R, and Q324K were more prevalent in African PfCSP, while D356N was more prevalent in South American and African PfCSP. Meanwhile, A361E was more prevalent in Asian, Oceanian, and South American PfCSP than in African PfCSP.

### Nucleotide diversity and natural selection of the C-terminal non-repeat region in Myanmar PfCSP

The nucleotide diversity and genetic differentiation were analysed in the C-terminal non-repeat region in Myanmar PfCSP. The average number of nucleotide differences (*K*) in this region was 0.92. The overall haplotype diversity (Hd) and nucleotide diversity (π) in this region were estimated to be 0.329 ± 0.072 and 0.004 ± 0.003, respectively. In order to investigate whether natural selection has contributed to the diversity in C-terminal non-repeat region in Myanmar PfCSP, the value of dN–dS for this region was analysed. The estimated value of dN–dS was found to be 0.005 ± 0.003 (Table 1), suggesting that this region may influenced by a positive natural selection. Tajima’s D test was also performed to further analyse the natural selection in the C-terminal non-repeat region in Myanmar PfCSP. Tajima’s D value was found to be 0.764 (*P* > 0.1) (Table 1). The Fu and Li’s D and F values were also positive at 0.889 (*P* > 0.1) and 0.992 (*P* > 0.1), respectively.

**Table 1:** Genetic polymorphism and tests of neutrality in C-terminal region of global PfCSP

Country	*n*	*K*	S	Total No. of mutations	H	Hd ± SD	π ± SD	dN–dS	Tajima’s D^*P*-*value*^	Fu and Li’s D^*P*-*value*^	Fu and Li’s F^*P*-*value*^
*Asia*											
Myanmar	51	0.92	3	3	3	0.329 ± 0.072	0.004 ± 0.003	0.005 ± 0.003	0.764^a^	0.889^a^	0.992^a^
Vietnam	23	4.39	14	16	23	1.000 ± 0.013	0.019 ± 0.001	0.024 ± 0.008	0.050^a^	0.862^a^	0.722^a^
Thailand	223	1.74	12	13	8	0.485 ± 0.039	0.009 ± 0.001	0.011 ± 0.004	− 0.483^a^	1.475^d^	0.886^a^
Philippines	42	5.79	18	19	9	0.821 ± 0.041	0.028 ± 0.001	0.033 ± 0.009	1.019^a^	1.649^b^	1.701^c^
Iran	21	0.93	2	2	2	0.467 ± 0.075	0.004 ± 0.001	0.006 ± 0.004	1.564^a^	0.858^a^	1.205^a^
India	15	3.71	18	19	13	0.981 ± 0.031	0.019 ± 0.004	0.023 ± 0.007	− 1.486^a^	− 1.173^a^	− 1.448^a^
Total	375	2.73	25	29	38	0.653 ± 0.027	0.013 ± 0.001	0.016 ± 0.005	− 1.032^a^	− 0.235^a^	− 0.698^a^
*Oceania*											
PNG	94	1.17	5	5	4	0.532 ± 0.034	0.006 ± 0.001	0.007 ± 0.004	0.419^a^	− 0.012^a^	0.151^a^
Vanuatu	116	0.55	2	2	2	0.276 ± 0.046	0.003 ± 0.001	0.003 ± 0.002	0.706^a^	0.674^a^	0.801^a^
Solomon Islands	51	0.83	4	4	3	0.397 ± 0.066	0.004 ± 0.0007	0.005 ± 0.003	− 0.1426^a^	− 1.209^a^	− 1.029^a^
Total	261	1.09	5	5	4	0.522 ± 0.012	0.005 ± 0.0002	0.007 ± 0.004	0.626^a^	− 0.214^a^	0.087^a^
*Africa*											
Kenya	18	5.83	17	20	12	0.922 ± 0.051	0.028 ± 0.003	0.035 ± 0.011	0.010^a^	0.295^a^	0.247^a^
Cameroon	9	4.56	12	15	7	0.944 ± 0.07	0.022 ± 0.005	0.028 ± 0.009	− 0.842^a^	− 0.689^a^	− 0.813^a^
Gambia	44	5.83	18	21	20	0.944 ± 0.019	0.028 ± 0.002	0.036 ± 0.009	0.677^a^	1.006^a^	1.068^a^
Ghana	33	5.02	18	22	19	0.932 ± 0.028	0.024 ± 0.003	0.031 ± 0.009	− 0.257^a^	0.474^a^	0.278^a^
Tanzania	60	6.45	22	26	30	0.969 ± 0.008	0.031 ± 0.001	0.039 ± 0.011	0.499^a^	0.852^a^	0.863^a^
Senegal	11	4.54	10	12	9	0.964 ± 0.051	0.019 ± 0.002	0.024 ± 0.007	0.475^a^	1.149^a^	1.106^a^
Total	175	21.76	150	169	58	0.968 ± 0.004	0.105 ± 0.021	0.169 ± 0.055	− 0.831^a^	2.648^b^	1.205^a^
*South America*											
Venezuela	10	6.44	14	16	7	0.911 ± 0.077	0.031 ± 0.003	0.034 ± 0.014	0.645^a^	0.799^a^	0.857^a^
Brazil	42	2.33	8	8	3	0.459 ± 0.08	0.011 ± 0.002	0.014 ± 0.005	0.721^a^	1.309^d^	1.317^a^
Total	52	3.74	16	18	9	0.630 ± 0.065	0.018 ± 0.002	0.022 ± 0.007	− 0.188^a^	0.431^a^	0.257^a^

*PNG* Papua New Guinea, *n* number of sequences analysed, *S* number of Segregating sites, *K* average number of nucleotide differences, *H* number of Haplotypes, *Hd* haplotype diversity, *π* observed average pairwise nucleotide diversity, *dN* rate of non-synonymous mutations, *dS* rate of synonymous mutations, *SD* standard deviation

^a^*P *> 0.1; ^b^ *P* < 0.02; ^c^ *P* < 0.05; ^d^ 0.05 < *P* < 0.1

### Nucleotide diversity, natural selection, and recombination of the C-terminal non-repeat region among global PfCSP

**Fig. 5:**
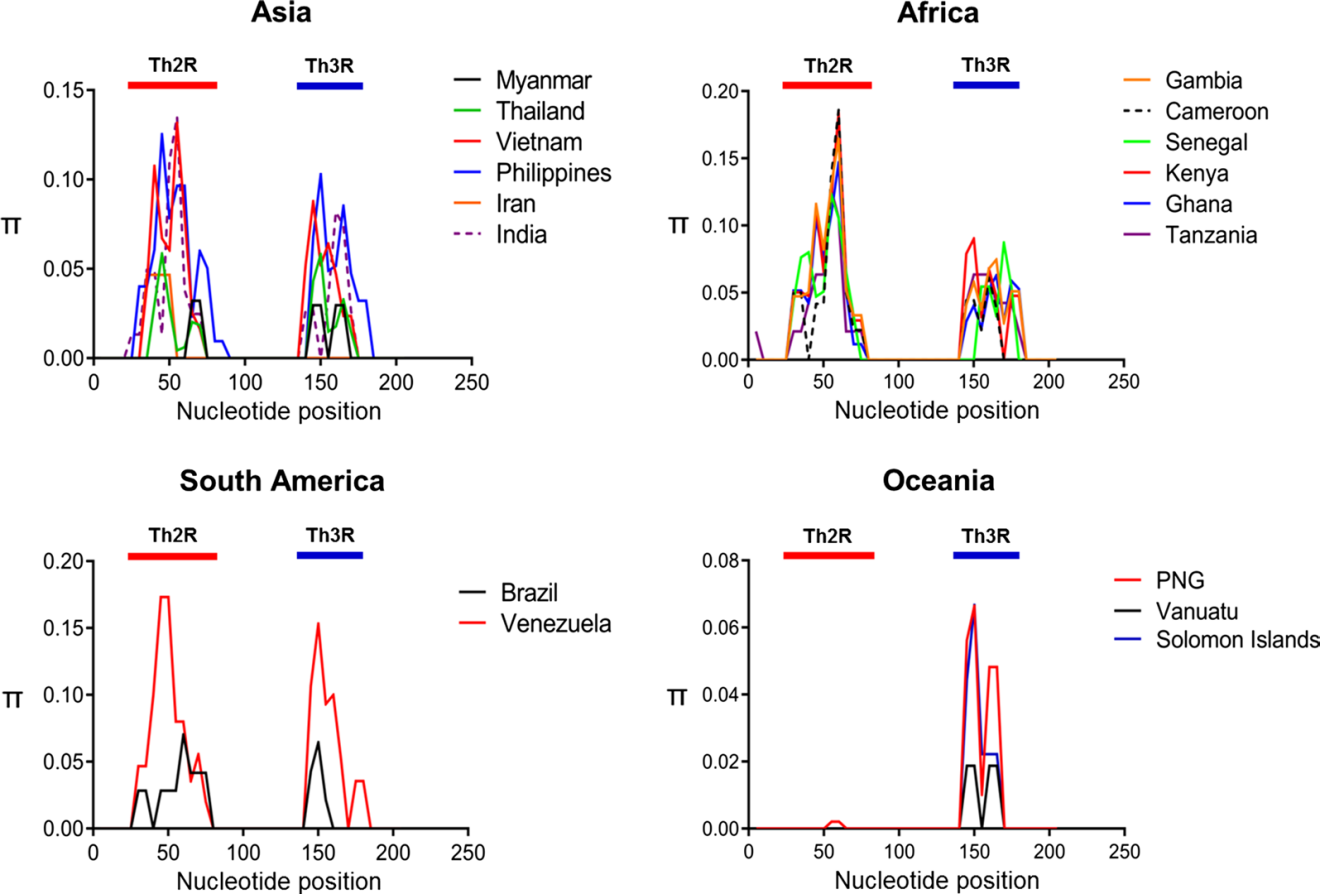
Nucleotide diversity of C-terminal non-repeat region among global PfCSP. Sliding window analysis presented nucleotide diversity (π) values in global PfCSP sequences. The values of nucleotide diversity per site (π) were calculated using the DnaSP with a window length of 10 bp and step size of 5 bp. *PNG* Papua New Guinea

Genetic diversity in the C-terminal non-repeat region among global PfCSP was analysed in order to assess the extent of genetic diversity between the populations (Table 1). The *K* value in African PfCSP (21.76) was higher than that in South American (3.74), Asian (2.73), and Oceanian (1.09) PfCSP. The greatest nucleotide diversity was observed in African PfCSP (π = 0.105 ± 0.021) followed by South American PfCSP (π = 0.018 ± 0.002), Asian PfCSP (π = 0.013 ± 0.001), and Oceanian PfCSP (π = 0.005 ± 0.0002). The dN–dS values for all global PfCSP were estimated to be positive, suggesting that positive natural selection may occur in the C-terminal non-repeat region of global PfCSP, but this trend was not statistically significant. Negative values of Tajima’s D were identified in the C-terminal non-repeat region of South American PfCSP (− 0.188, *P* > 0.1), African PfCSP (− 0.831, *P* > 0.1), and Asian PfCSP (− 1.032, *P* > 0.1), indicating that they were under purifying selection. Meanwhile, the C-terminal non-repeat region of Oceanian PfCSP (0.626, *P* > 0.1) showed positive Tajima’s D values, suggesting the effects of balance selection on the population. Sliding window plot analysis (window length of 10 bp and step size of 5 bp) in global PfCSP showed that global PfCSP shared highly similar patterns of nucleotide diversity across the region. Nucleotide diversity peaked at the Th2R and Th3R T-cell epitopes, although the values of π were slightly different within and between PfCSP populations according to geographical origin (Fig. 5). Interestingly, only a single major peak of nucleotide diversity was identified at the Th3R epitopes in Oceanian PfCSP. Recombination events were predicted in Myanmar and global PfCSP. The recombination parameters expected in PfCSP-differed by geographical population, but a possible recombination event has been suggested in global PfCSP (Table 2). High Rm values were predicted for African PfCSP, while lower levels of Rm were identified in PfCSP from other geographical areas.

**Table 2:** Recombination events in global PfCSP

Country	Ra	Rb	Rm
Myanmar	0.000	0.001	0
Vietnam	0.504	116	3
Thailand	0.000	0.001	2
Philippines	0.082	16.9	1
India	0.000	0.001	0
Iran	0.043	8.2	2
PNG	0.024	4.9	1
Solomon Islands	0.000	0.001	0
Vanuatu	0.000	0.001	0
Ghana	0.071	19.5	6
Tanzania	0.171	35.1	6
Cameroon	0.077	15.9	2
Senegal	0.172	41.2	2
Kenya	0.110	22.7	6
Gambia	0.116	23.9	5
Brazil	0.000	0.001	0
Venezuela	0.1403	28.9	2

The R and Rm were estimated excluding the sites containing alignment gaps or those segregating for three nucleotides

*Ra* recombination parameter between adjacent sites, *Rb* recombination parameter for entire gene, *Rm* minimum number of recombination events between adjacent sites, *PNG* Papua New Guinea

### Haplotype network analysis of PfCSP among global *P. falciparum* isolates

**Fig. 6:**
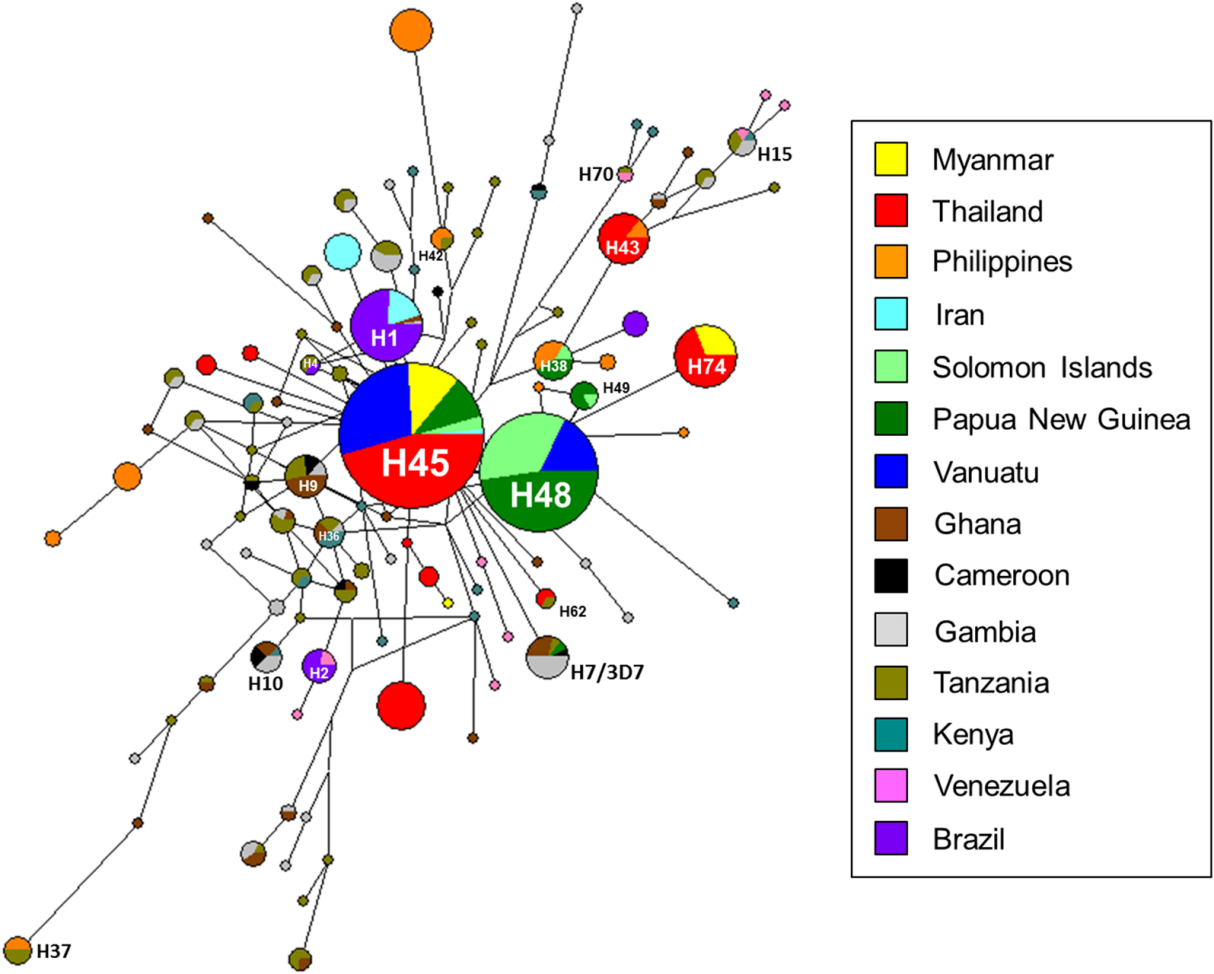
Haplotype network analysis of global PfCSP. Haplotype network of the PfCSP was constructed using the NETWORK program version 5.0.0.3 with the Median Joining algorithm. The network showed 103 haplotypes found in 817 global PfCSP sequences. Branch lengths were the proportion of divergence. The size of each node indicated the proportion of the total haplotype frequencies. The color of each node corresponded to a different geographic region

A haplotype network was constructed in order to analyse the relationships between and among PfCSP from global *P. falciparum* isolates. Indian, Vietnamese, and Senegal PfCSP sequences were precluded, as these sequences were not cover full-length sequences. A total of 103 distinct haplotypes were identified in the 817 global PfCSP sequences analysed (Fig. 6). Forty-eight haplotypes (46.6%) were shared by PfCSP sequences from at least two different countries, while 55 haplotypes with a frequency of 53.4% were limited to singleton. The haplotype prevalence ranged from 0.12% to 41.86%. Haplotype 45 (H45) was the most prevalent haplotype with a frequency of 41.86% and was occupied by Asian and Oceanian PfCSP. The two haplotypes of H48 and H49 were shared only with Oceanian PfCSP. Meanwhile, the two haplotypes of H43 and H74 were composed only of Asian PfCSP. Only nine haplotypes (H1, H4, H7, H15, H37, H42, H45, H62, and H70) were made by inter-continental PfCSP. No haplotypes were identified to be mixed haplotypes shared among populations from all four continents. Most singletons were mainly identified among African PfCSP, suggesting that high genetic polymorphisms occurred in the African population. Haplotypes from Venezuela PfCSP were closely linked to African PfCSP, suggesting genetic closeness between the two populations. The haplotype identical to the 3D7 sequence (XM_001351086) was haplotype 7 (H7), which occupied 1.71% of frequency and was mainly shared by African PfCSP.

## Discussion

Diverse kinds of *P. falciparum* antigens have been extensively studied as candidate antigens for a malaria vaccine. However, genetic and antigenic variations in vaccine candidates in the global *P. falciparum* population have been immense challenges in developing an effective malaria vaccine and to certify the efficacy of the vaccine. Therefore, understanding the genetic nature and antigenic variation of vaccine candidate antigens among global *P. falciparum* populations is important since this can provide potential rejoinders on the effects of genetic diversity in the global population for vaccine efficacy and valuable information for designing optimal vaccine formulation [27]. PfCSP is a leading candidate for a malaria vaccine and recent Phase III RTS,S vaccine trials resulted in significant reduction rates in clinical malaria [5, 6]. However, the PfCSP antigen formulated in RTS,S is a single variant, and therefore the impact of natural genetic variation in the global PfCSP population on vaccine efficacy remains unclear. In this study, the genetic polymorphism and natural selection in the Myanmar PfCSP and global PfCSP populations were comprehensively analysed.

Myanmar PfCSP had a largely well-conserved N-terminal region, which coincided with PfCSP populations from other geographical areas [18, 27–30]. A few amino acid polymorphisms were identified in global PfCSP populations, but A98G was the only commonly identified amino acid change in global PfCSP N-terminal region, although its frequency differed by country. The most important polymorphic characteristic identified in Myanmar and global PfCSP was a 19 amino acid length insertion (NNGDNGREGKDEDKRDGNN) in the middle of this region. This insertion was identified in all global PfCSP enrolled in this study with the only exception being Indian PfCSP, but the frequency of this insertion varied with PfCSP populations from different geographical regions. The N-terminal region of PfCSP is known to play an essential role in the invasion process of sporozoites to hepatic cells by mediating or facilitating the interaction between sporozoites and host cells [30–32]. A monoclonal antibody that binds to a linear epitope, ^81^EDNEKLRKPKH^91^, in the N-terminal region of PfCSP effectively neutralizes sporozoite infectivity in vivo, suggesting a critical role for this epitope in sporozoite infectivity to hepatocytes [33]. The functional significance of the 19 amino acids insertion in PfCSP N-terminal region is currently unclear. However, considering that this insertion is essentially located in the front of the ^81^EDNEKLRKPKH^91^ linear epitope, and that global PfCSP, except for the Indian population, had the insertion, a study aimed at understanding the role and evolutionary implication of this insertion is warranted. Most amino acid polymorphisms identified in the N-terminal region of global PfCSP was located in the predicted T-cell epitope region (^84^EKLRKPKHKKLKQPADGNPDP^104^), indicating that this region is under host immune responses. The N-terminal region of PfCSP has been largely neglected as a potential vaccine target in spite of being a target of inhibitory antibodies and protective T cell responses. The functional importance of the N-terminal region in protective immunity has been demonstrated. Polypeptides flanking the PfCSP N-terminal region evoked the production of inhibitory antibodies for hepatocyte invasion by sporozoites, and these polypeptides are likely to render partial protective immunity in people residing in malaria-endemic regions [34]. A recent study also suggested that most of the effective antibodies that potently inhibit malaria infection bind not only to the repeat region, but also to a portion of N-terminal junction of PfCSP [35]. These collectively highlighted the potential of the N-terminal region of PfCSP as a part of PfCSP-based vaccine constructs for malaria vaccine formulation. The low genetic polymorphic nature in the N-terminal region of global PfCSP also supports the notion that the region can be an attractive component of PfCSP-based vaccine.

The central repeat region of PfCSP has been recognized to play crucial roles in sporozoite formation and development [36]. It has been postulated that the genetic diversity of this region may be maintained by balancing selection, mainly affected by host’s immune responses [33]. Differing numbers of tetrapeptide repeats have been identified as an important source of genetic polymorphism in PfCSP. As expected, high levels of genetic polymorphisms due to different numbers of repeats were identified in the central repeat region of Myanmar PfCSP, which resulted in 14 different haplotypes. Interestingly, two novel repeats, NANS and NTNP, were identified in two haplotypes of Myanmar PfCSP, although their frequencies were low. Numerous variant forms of repeats including NVVP, NAKP, NAHP, NAIP, NVNP, NANL, NVAD, NPNP, NADP, KANP, and SANP have been reported in global PfCSP [18], but the effect of these variations is still not clearly understood. The number of repeats in the central repeat region is known to affect PfCSP stability. The stability of the type-β turn structure increases with the number of repeats [27]. Myanmar PfCSP had a high number of tetrapeptide repeats in the central repeat region, as 86.3% of Myanmar PfCSP had a number of repeats ranging from 40 to 43. Comparative analysis of the number of NANP repeats in Myanmar PfCSP and global PfCSP suggested a differing distribution of the repeats according to geographical origin, with the highest in Asian PfCSP (40–43) and the lowest in African and South American PfCSP (36–37). These suggested that PfCSP may have evolved separately, probably by evolutionary force in order to maintain or enhance protein stability, or to evade host immune response, in different geographical origin *P. falciparum* populations, resulting in the differing number of tetrapeptide repeats in the global PfCSP population. The RTS,S, the current malaria vaccine, is composed of 19 NANP tetrapeptide repeats and C-terminal T cell-epitope that are linked to the Hepatitis B surface antigen [37]. To date, there has been no direct evidence indicating that different numbers of repeats can affect the efficiency of RTS,S. However, considering that highly heterogenous numbers of repeats are maintained in the natural PfCSP population, studies evaluating the effects of polymorphic nature in the central repeat region to RTS,S vaccine efficacy are necessary.

The C-terminal non-repeat region of Myanmar PfCSP displayed limited diversity with only three differing haplotypes among 51 Myanmar PfCSP sequences, coinciding with the previous reports on PfCSP from different geographical origins [29]. Haplotype 3, which had KHIEQYLKKIQNSL and NKPKDELDYEND in the Th2R and Th3R regions, was the most prevalent haplotype found in Myanmar PfCSP. This allelic variant was also detected at a high frequency in Asian PfCSP populations [18, 28, 38, 39]. The overall values for haplotype diversity (H) and nucleotide diversity (π) for PfCSP C-terminal region were higher in African PfCSP than in PfCSP from other continents, indicating that African PfCSP had higher level of genetic diversity. Comparative sliding window plot analysis of π in the C-terminal region of global PfCSP revealed similar patterns of nucleotide diversity across the region. Asian PfCSP, African PfCSP, and South American PfCSP displayed relatively similar patterns of π with two peaks at the Th2R and Th3R regions, suggesting that the genetic variations were mainly concentrated at these regions. However, differences were also found between or among PfCSP from different geographical areas. A greater π value was identified at the Th2R region than the Th3R region in Asian, African, and South American PfCSP. Meanwhile, Oceanian PfCSP revealed only a major peak of π value at the Th3R region. Polymorphisms in the Th3R region have been demonstrated as being associated with HLA binding and cytotoxic T cell reactivity [40, 41], thus these polymorphisms may assist parasites in escaping the host immune pressure. Natural selection analysis of global PfCSP C-terminal region suggests that this region is likely to be under natural selection which may maintain or generate genetic diversity in the global PfCSP population. The dN–dS values for Myanmar PfCSP and global PfCSP were positive, implying that balancing selection might act in this region. The values of Tajima’s D and Fu and Li’s D and F revealed complicated patterns that were distinct between or among global PfCSP. These results suggested that global PfCSP was under a complicated influence of natural selection, in which either positive selection or purifying selection might have occurred in the population, depending on the geographical origin. Possible recombination events in the global PfCSP C-terminal region were also predicted. Higher values of recombination events were found in African PfCSP than in PfCSP from other geographical areas, suggesting that African PfCSP might allow for more opportunity for inter- or intra-allelic recombination than other geographical PfCSP. This might be due to the high multiclonal infection rate of the parasite as well as subsequent cross fertilization and active recombination in mosquitoes in Africa. Interestingly, non-neglectable recombination parameters with a high haplotype diversity were predicted in Vietnamese PfCSP. Compared to the values for recombination parameters and haplotype diversity of other Asian PfCSP populations, these were extremely high in Vietnamese PfCSP.

Considering that Vietnam is a hypoendemic country with a low malaria transmission rate, the reason why Vietnamese PfCSP showed high recombination event and haplotype diversity is unclear and it should be elucidated further. Collectively, the results of genetic diversity analysis in the C-terminal region of global PfCSP suggested that global PfCSP showed limited genetic diversity in the region. However, the genetic diversity pattern of the PfCSP C-terminal region differed slightly according to different geographical origins. Complicated natural selection acts on the global PfCSP C-terminal region, which produces genetic diversity of the region in global PfCSP. Recombination may also contribute to the genetic diversity of global PfCSP, although the recombination parameters differed by geographical origins. These genetic polymorphisms in the C-terminal region of global PfCSP suggest that more concern is required for design formulation of PfCSP-based vaccine.

Haplotype network analysis of 817 global PfCSP sequences indicated that Asian and Oceanian PfCSP formed limited numbers of clusters. Meanwhile, African PfCSP showed highly-branched and complicated patterns of haplotype diversity. No haplotype was identified that fully covers PfCSP from all of the geographic regions analysed in this study. Most singletons were mainly occupied by African PfCSP, supporting the notion that African PfCSP had higher genetic diversity than PfCSP from other geographical regions. The current RTS,S recombinant vaccine was constructed with PfCSP of *P. falciparum* NF54/3D7 strain [42]. Haplotype 7 with a frequency of 1.71%, which was shared by African PfCSP, was identical to 3D7 PfCSP. Many studies on evaluating the effectiveness and safety of RTS,S have been performed in Africa [5, 43–46], and it has been suggested that RTS,S is likely to be effective, at least in Africa. However, its efficacy worldwide may be challenging. As presented in this study, genetic heterogeneity of the PfCSP regions included in RTS,S, as well as the complicated haplotype diversity of PfCSP between and among global PfCSP, suggest that more attention is necessary toward developing a PfCSP-based vaccine, and a new approach for RTS,S that is effective in a variety of areas should be considered. If it is difficult to develop effective vaccine that works against global malaria populations, the development of an individual vaccine that works in particular malaria transmission areas by including genotypes prevalent in the geographical regions can also be considered. For example, considering that H45 and H48 are the most prevalent haplotypes of PfCSP in the Asian and Oceanian PfCSP populations, these haplotypes could be considered in designing a PfCSP-based vaccine for Asian and Oceanian countries.

The limitation of this study is that Myanmar PfCSP sequences analysed in this study were from *P. falciparum* isolates that collected in restricted areas of Myanmar. Therefore, nation-wide analysis of PfCSP in *P. falciparum* isolates collected from different regions of Myanmar is needed to clearly understand the overall genetic diversity and population structure of Myanmar PfCSP. Further examination of PfCSP nucleotide and amino acid variations in diverse PfCSP populations with a larger number of global PfCSP sequences would be also necessary to better understand the polymorphic nature of PfCSP.

## Conclusions

PfCSP is a main component of RST,S, the most advanced malaria vaccine currently, but the impact of natural genetic variations in the global PfCSP population on the efficacy of RTS,S remains unclear. Analysis of genetic diversity and natural selection of global PfCSP population suggested that the central repeat region was highly polymorphic in global PfCSP. Meanwhile, the N-terminal and C-terminal non-repeat regions revealed limited polymorphism, but differences were also identified between or among global PfCSP according to the different geographical origins. In particular, a high level of nucleotide diversity was identified in the Th2R and Th3R regions of the PfCSP C-terminal region. Complicated natural selection and recombination event were predicted in the global PfCSP C-terminal region, which may be major driving forces for maintaining and producing genetic diversity of the region in global PfCSP. The geographic patterns of genetic differentiation as well as the points of evidence for natural selection and recombination suggest that the functional consequences of the polymorphism should be considered for a vaccine based on PfCSP. The results of this study not only contribute insight into the genetic nature of Myanmar PfCSP, but also aid the understanding of genetic diversity of global PfCSP, and may provide valuable information for the development of an effective vaccine based on PfCSP. The findings of this study also warrant continuous monitoring of genetic diversity of PfCSP in the global population to better understand the polymorphic nature and evolutionary aspect of PfCSP in global *P. falciparum* population.

## Additional file

**Additional file 1: Table S1.** Global PfCSP sequences analysed in this study.

## Abbreviations

dS: rates of synonymous substitutions
dN: rate of non-synonymous substitutions
H: haplotypes
Hd: haplotype diversity
*K*: average number of pair-wise nucleotide differences within the population
PCR: polymerase chain reaction
PfCSP: circumsporozoite surface protein of *Plasmodium falciparum*
PNG: Papua New Guinea
Rm: minimum number of recombination events between adjacent polymorphic sites
S: segregating sites
π: nucleotide diversity

## References

[1] World Health Organization (2017). World malaria report 2017.

[2] World Health Organization (2017). Malaria in the Greater Mekong Subregion: Regional and country profiles.

[3] World Health Organization (2015). Strategy for Malaria Elimination in the Greater Mekong Subregion (2015–2030).

[4] Kang JM, Cho PY, Moe M, Lee J, Jun H, Lee HW (2017). Comparison of the diagnostic performance of microscopic examination with nested polymerase chain reaction for optimum malaria diagnosis in Upper Myanmar. Malar J..

[5] Agnandji ST, Lell B, Soulanoudjingar SS, Fernandes JF, Abossolo BP, Conzelmann C (2011). First results of phase 3 trial of RTS,S/AS01 malaria vaccine in African children. N Engl J Med.

[6] Agnandji ST, Lell B, Fernandes JF, Abossolo BP, Methogo BG, Kabwende AL (2012). A phase 3 trial of RTS,S/AS01 malaria vaccine in African infants. N Engl J Med.

[7] Pinzon-Ortiz C, Friedman J, Esko J, Sinnis P (2001). The binding of the circumsporozoite protein to cell surface heparan sulfate proteoglycans is required for *Plasmodium* sporozoite attachment to target cells. J Biol Chem.

[8] Rathore D, Sacci JB, de la Vega P, McCutchan TF (2002). Binding and invasion of liver cells by *Plasmodium falciparum* sporozoites. Essential involvement of the amino terminus of circumsporozoite protein. J Biol Chem..

[9] Coppi A, Natarajan R, Pradel G, Bennett BL, James ER, Roggero MA (2011). The malaria circumsporozoite protein has two functional domains, each with distinct roles as sporozoites journey from mosquito to mammalian host. J Exp Med.

[10] Egan JE, Hoffman SL, Haynes JD, Sadoff JC, Schneider I, Grau GE (1993). Humoral immune responses in volunteers immunized with irradiated *Plasmodium falciparum* sporozoites. Am J Trop Med Hyg.

[11] Nardin EH, Nussenzweig RS, McGregor IA, Bryan JH (1979). Antibodies to sporozoites: their frequent occurrence in individuals living in an area of hyperendemic malaria. Science.

[12] Calvo-Calle JM, de Oliveira GA, Clavijo P, Maracic M, Tam JP, Lu YA (1993). Immunogenicity of multiple antigen peptides containing B and non-repeat T cell epitopes of the circumsporozoite protein of *Plasmodium falciparum*. J Immunol..

[13] Hughes AL (1991). Circumsporozoite protein genes of malaria parasites (Plasmodium spp.): Evidence for positive selection on immunogenic regions. Genetics..

[14] Waitumbi JN, Anyona SB, Hunja CW, Kifude CM, Polhemus ME, Walsh DS (2009). Impact of RTS,S/AS02(A) and RTS,S/AS01(B) on genotypes of *P. falciparum* in adults participating in a malaria vaccine clinical trial. PLoS ONE..

[15] Bailey JA, Mvalo T, Aragam N, Weiser M, Congdon S, Kamwendo D (2012). Use of massively parallel pyrosequencing to evaluate the diversity of and selection on *Plasmodium falciparum* csp T-cell epitopes in Lilongwe, Malawi. J Infect Dis..

[16] Neafsey DE, Schaffner SF, Volkman SK, Park D, Montgomery P, Milner DA (2008). Genome-wide SNP genotyping highlights the role of natural selection in *Plasmodium falciparum* population divergence. Genome Biol.

[17] Manske M, Miotto O, Campino S, Auburn S, Almagro-Garcia J, Maslen G (2012). Analysis of *Plasmodium falciparum* diversity in natural infections by deep sequencing. Nature.

[18] Zeeshan M, Alam MT, Vinayak S, Bora H, Tyagi RK, Alam MS (2012). Genetic variation in the *Plasmodium falciparum* circumsporozoite protein in India and its relevance to RTS,S malaria vaccine. PLoS One..

[19] Snounou G, Singh B (2002). Nested PCR analysis of Plasmodium parasites. Methods Mol Med.

[20] Librado P, Rozas J (2009). DnaSP v5: a software for comprehensive analysis of DNA polymorphism data. Bioinformatics.

[21] Tamura K, Stecher G, Peterson D, Filipski A, Kumar S (2013). MEGA6: molecular evolutionary enetics analysis version 6.0. Mol Biol Evol..

[22] Nei M, Gojobori T (1986). Simple methods for estimating the numbers of synonymous and nonsynonymous nucleotide substitutions. Mol Biol Evol.

[23] Tajima F (1989). Statistical method for testing the neutral mutation hypothesis by DNA polymorphism. Genetics.

[24] Fu YX, Li WH (1993). Statistical tests of neutrality of mutations. Genetics.

[25] Bandelt HJ, Forster P, Rohl A (1999). Median-joining networks for inferring intraspecifc phylogenies. Mol Biol Evol.

[26] Sidjanski SP, Vanderberg JP, Sinnis P (1997). *Anopheles stephensi* salivary glands bear receptors for region I of the circumsporozoite protein of *Plasmodium falciparum*. Mol Biochem Parasitol.

[27] Escalante AA, Grebert HM, Isea R, Goldman IF, Basco L, Magris M (2002). A study of genetic diversity in the gene encoding the circumsporozoite protein (CSP) of *Plasmodium falciparum* from different transmission areas-XVI. Asembo Bay Cohort Project. Mol Biochem Parasitol..

[28] Putaporntip C, Jongwutiwes S, Hughes AL (2009). Natural selection maintains a stable polymorphism at the circumsporozoite protein locus of *Plasmodium falciparum* in a low endemic area. Infect Genet Evol..

[29] Zakeri S, Avazalipoor M, Mehrizi AA, Djadid ND, Snounou G (2007). Restricted T-cell epitope diversity in the circumsporozoite protein from *Plasmodium falciparum* populations prevalent in Iran. Am J Trop Med Hyg.

[30] Plassmeyer ML, Reiter K, Shimp RL, Kotova S, Smith PD, Hurt DE (2009). Structure of the *Plasmodium falciparum* circumsporozoite protein, a leading malaria vaccine candidate. J Biol Chem.

[31] Rathore D, Nagarkatti R, Jani D, Chattopadhyay R, de la Vega P, Kumar S (2005). An immunologically cryptic epitope of *Plasmodium falciparum* circumsporozoite protein facilitates liver cell recognition and induces protective antibodies that block liver cell invasion. J Biol Chem.

[32] Ancsin JB, Kisilevsky R (2004). A binding site for highly sulfated heparan sulfate is identified in the N terminus of the circumsporozoite protein: significance for malarial sporozoite attachment to hepatocytes. J Biol Chem.

[33] Conway DJ (1997). Natural selection on polymorphic malaria antigens and the search for a vaccine. Parasitol Today..

[34] Bongfen SE, Ntsama PM, Offner S, Smith T, Felger I, Tanner M (2009). The N-terminal domain of *Plasmodium falciparum* circumsporozoite protein represents a target of protective immunity. Vaccine..

[35] Tan J, Sack BK, Oyen D, Zenklusen I, Piccoli L, Barbieri S (2018). A public antibody lineage that potently inhibits malaria infection through dual binding to the circumsporozoite protein. Nat Med.

[36] Ferguson DJ, Balaban AE, Patzewitz EM, Wall RJ, Hopp CS, Poulin B (2014). The repeat region of the circumsporozoite protein is critical for sporozoite formation and maturation in *Plasmodium*. PLoS ONE.

[37] Garçon N, Heppener DG, Cohen J (2003). Development of RTS,S/AS02: a purified subunit-based malaria vaccine candidate formulated with a novel adjuvant. Expert Rev Vaccines..

[38] Kumkhaek C, Phra-ek K, Renia L, Singhasivanon P, Looareesuwan S, Hirunpetcharat C (2005). Are extensive T cell epitope polymorphisms in the *Plasmodium falciparum* circumsporozoite antigen, a leading sporozoite vaccine candidate, selected by immune pressure?. J Immunol..

[39] Bhattacharya PR, Bhatia V, Pillai CR (2006). Genetic diversity of T-helper cell epitopic regions of circumsporozoite protein of *Plasmodium falciparum* isolates from India. Trans R Soc Trop Med Hyg.

[40] Udhayakumar V, Ongecha JM, Shi YP, Aidoo M, Orago AS, Oloo AJ (1997). Cytotoxic T cell reactivity and HLA-B35 binding of the variant *Plasmodium falciparum* circumsporozoite protein CD8+ CTL epitope in naturally exposed Kenyan adults. Eur J Immunol.

[41] Hill AV, Elvin J, Willis AC, Aidoo M, Allsopp CE, Gotch FM (1992). Molecular analysis of the association of HLA-B53 and resistance to severe malaria. Nature.

[42] Gordon DM, McGovern TW, Krzych U, Cohen JC, Schneider I, LaChance R (1995). Safety, immunogenicity, and efficacy of a recombinantly produced *Plasmodium falciparum* circumsporozoite protein-hepatitis B surface antigen subunit vaccine. J Infect Dis.

[43] The RTS,S Clinical Trial Partnership (2015). Efficacy and safety of RTS,S/AS01 malaria vaccine with or without a booster dose in infants and children in Africa: final results of a phase 3, individually randomised, controlled trial. Lancet..

[44] Otieno L, Oneko M, Otieno W, Abuodha J, Owino E, Odero C (2016). Safety and immunogenicity of RTS,S/AS01 malaria vaccine in infants and children with WHO stage 1 or 2 HIV disease: a randomised, double-blind, controlled trial. Lancet Infect Dis..

[45] The RTS,S Clinical Trials Partnership (2014). Efficacy and Safety of the RTS,S/AS01 malaria vaccine during 18 months after vaccination: A phase 3 randomized, controlled trial in children and young infants at 11 African sites. PLoS Med..

[46] Kaslow DC, Biernaux S (2015). RTS,S: toward a first landmark on the malaria vaccine technology roadmap. Vaccine..

