# Supplementary material for: Genetic polymorphism and natural selection of circumsporozoite surface protein in Plasmodium falciparum field isolates from Myanmar
Source: Malar J. 2018 Oct 12;17:361. doi: 10.1186/s12936-018-2513-0 (PMC6186114; doi:10.1186/s12936-018-2513-0)
Supplement: Supplementary file 1 — Additional file 1: Table S1. Global PfCSP sequences analysed in this study. [file 12936_2018_2513_MOESM1_ESM.docx]

| **Country** | **Accession number** | ***n*** |
| --- | --- | --- |
| Thailand | FJ232142− FJ232364 | 223 |
| Philippines | AB502965− AB503006 | 42 |
| Vietnam | DQ193573− DQ193595 | 23 |
| Iran | DQ521732− DQ521752 | 21 |
| India | HM582036− HM582081, HM756094− HM756103, HM756105− HM756109 | 61 |
| Papua New Guinea | AB503007− AB503100 | 94 |
| Vanuatu | AB715520− AB715635 | 116 |
| Solomon Islands | AB503101− AB503151 | 51 |
| Kenya | AF540443, AF540447, AF540448, AF540450, AF540451, AF540455− AF540457, AF540461− AF540463, AF540465, AF540467, AF540473− AF540477 | 18 |
| Cameroon | AF540444− AF540446, AF540449, AF540452, AF540453, AF540454, AF540468, AF540480 | 9 |
| Gambia | AY878598− AY878641 | 44 |
| Ghana | AB502856− AB502888 | 33 |
| Tanzania | AB502796− AB502855 | 60 |
| Senegal | AJ269948, AJ269961− AJ269970 | 11 |
| Venezuela | AF540458− AF540460, AF540464, AF540466, AF540469, AF540470, AF540471, AF540478, AF540479 | 10 |
| Brazil | AB503152− AB503193 | 42 |

**Table S1** Global PfCSP sequences analysed in this study
